# Metabolomic and Proteomic Profiling of Women with Gestational Diabetes Mellitus

**DOI:** 10.3390/nu18121971

**Published:** 2026-06-18

**Authors:** Anna Maria Rzewuska-Fijałkowska, Tomasz Gęca

**Affiliations:** Department of Obstetrics and Pathology of Pregnancy, Medical University of Lublin, 20-081 Lublin, Poland

**Keywords:** gestational diabetes mellitus (GDM), metabolomics, proteomics, metabolic profiling, maternal health, omics technologies

## Abstract

Gestational diabetes mellitus (GDM), as one of the most common metabolic disorders occurring during pregnancy, represents a significant public health concern due to its rising prevalence and the numerous complications that can affect both the mother and the foetus. In recent years, there has been growing interest in the use of omics technologies, such as metabolomics and proteomics, in research on the pathogenesis and early detection of GDM. The aim of this paper was to summarise the current knowledge on metabolomic and proteomic changes observed in women with GDM and to assess the potential usefulness of these methods in identifying biomarkers of the disease. The narrative review was conducted in accordance with the PRISMA 2020 statement, using PubMed and Web of Science until 23 December 2025. The studies analysed show that GDM is associated with abnormalities in the metabolism of lipids, amino acids, carbohydrates and metabolites associated with the gut microbiota. The most commonly observed changes included: elevated levels of branched-chain amino acids, free fatty acids and purine metabolites, as well as changes in the metabolism of phospholipids and acylcarnitines. Multi-omics studies also indicate significant changes in plasma protein and lipid profiles. The data collected suggest that omics technologies may be a promising tool for identifying early biomarkers of GDM and for developing our understanding of the pathophysiological mechanisms of this condition. Nevertheless, further studies involving larger and more diverse patient populations are needed to confirm their diagnostic and clinical value.

## 1. Introduction

Gestational diabetes mellitus (GDM), as defined by the World Health Organization (WHO), is a disorder of carbohydrate tolerance that causes elevated blood glucose levels, which appears or is diagnosed for the first time during pregnancy [1]. It is one of the most common metabolic disorders during pregnancy, affecting 5.4% of all pregnant women in Europe, and its prevalence is steadily rising in line with the increase in obesity and metabolic disorders among women of reproductive age. The pathogenesis of GDM involves physiologically progressing insulin resistance induced by placental hormones, as well as a reduced pancreatic reserve, whose capacity is insufficient to meet the increased demand for insulin during pregnancy, particularly in the second trimester [2].

GDM is associated with an increased risk of perinatal complications, such as macro-somia and foetal fetopathy, preterm delivery, intrauterine foetal death, and increased peri-natal mortality in newborns. In the long term, it is also linked to the development of type 2 diabetes and cardiovascular diseases in mothers, as well as metabolic disorders in chil-dren [3]. Table 1 sets out the criteria used to diagnose GDM based on glucose levels during an oral glucose tolerance test (OGTT). For treatment purposes, there are two types of GDM: GDM1—dietary management and lifestyle changes are sufficient to achieve normogly-caemia; GDM2—insulin therapy is required to achieve normoglycaemia [4].

In recent years, there has been growing interest in the use of omics technologies, such as metabolomics, proteomics, lipidomics and multi-omics—research methods that integrate data from various biological levels, including genomics, transcriptomics and proteomics and metabolomics in research on GDM [5]. Metabolomics enables a comprehensive analysis of small-molecule metabolites in various types of biological samples (serum, plasma, urine, faeces, amniotic fluid), reflecting the body’s current metabolic state [6]. On the other hand, proteomics facilitates quantitative and qualitative profiling of proteins and their post-translational modifications, which play a key role in the regulation of hormonal signalling [7]. Both techniques, whether used in isolation or combined in multivariate analyses, enable the identification of biomarkers that may be capable of predicting GDM at an early stage, even before clinically detectable abnormalities in glucose metabolism become apparent.

The aim of this review is to present the current state of knowledge regarding the metabolomic and proteomic profiling of women with GDM, to discuss the most significant biomarkers identified and the potential pathophysiological mechanisms, and to assess the potential for the use of omics-based methods in the future diagnosis and early detection of GDM.

## 2. Methodology

**Figure 1:**
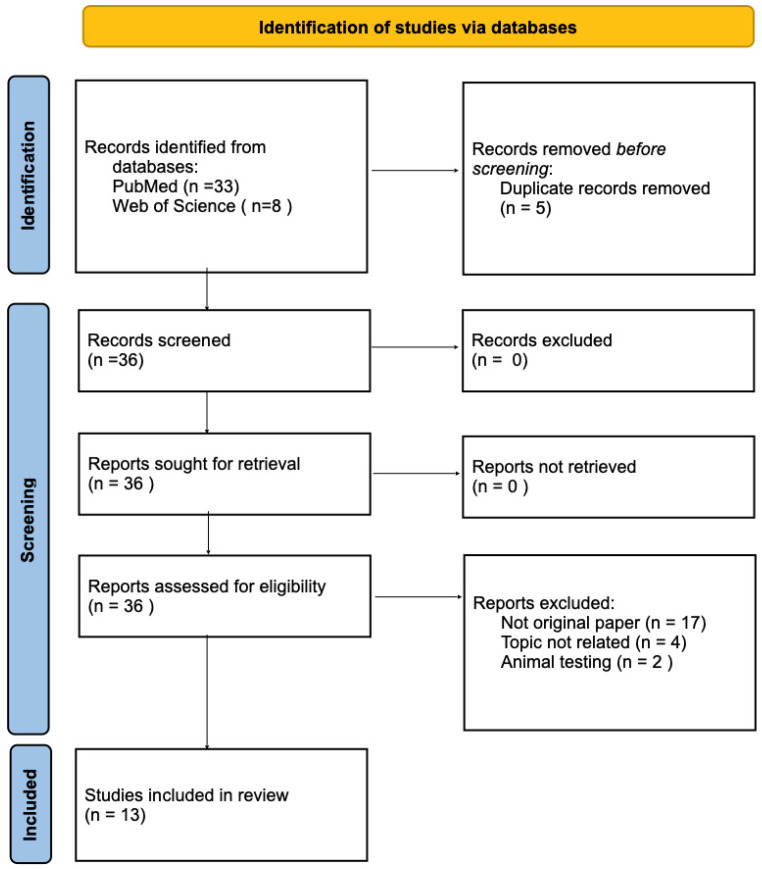
PRISMA diagram (figure created independently based on the PRISMA diagram [8]).

The narrative review was conducted in accordance with the PRISMA 2020 statement, using PubMed and https://www.webofscience.com/wos/woscc/basic-search (accessed on 1 November 2025) until 23 December 2025 [8]. The search terms were: (“gestational diabetes mellitus”) OR “gestational diabetes” OR GDM) AND (metabolomic OR metabolomics) AND (proteomic OR proteomics). This review includes only 13 original papers, written in English, conducted on a group of humans, with no use of animals, focusing on the topic of metabolomic and proteomic profiling of women with GDM (Figure 1).

## 3. Results

Between 2010 and 2013, a series of papers by Ana M. Gil et al. was published, which represents one of the first detailed analyses of metabolomic changes during the second trimester of pregnancy. Although the main aim of the study was to identify biomarkers of genetic disorders in the foetus, the results also provided data on early metabolic changes in women with GDM.

In the first study in this series, Graça et al. [9] analysed the metabolic profile of amniotic fluid collected between the 15th and 22nd week of pregnancy during amniocentesis. The analysis included both uncomplicated pregnancies and those complicated by various disorders, including GDM. Patients with GDM had a tendency toward increased glucose concentrations in the amniotic fluid, elevated lactate levels, and reduced concentrations of acetate, creatinine, formate, glutamate, glycine, proline, serine, and taurine. The authors emphasised that relatively small differences in concentrations between the study group and the control group may have been due to the fact that the samples were collected several weeks before the clinical diagnosis of GDM. This study was of crucial importance as it demonstrated that amniotic fluid, which directly reflects the intrauterine environment, can provide information on the early ‘metabolic programming’ of the foetus under the influence of the mother’s impaired glucose metabolism [9].

In a subsequent study, Díaz et al. extended their investigation to include an analysis of urine and plasma samples from pregnant women between 15 and 22 weeks of gestation [10]. In pregnant women, even before a diagnosis of GDM, increased urinary excretion of 3-hydroxyisovaleric acid and 2-hydroxyisobutyric acid was observed. Elevated levels of 3-hydroxyisovalerate may indicate biotin metabolism disorders and reduced activity of biotin-dependent enzymes, which may reflect early abnormalities in energy metabolism [11]. In turn, 2-hydroxyisobutyrate is associated with the metabolism of branched-chain amino acids (BCAAs) (valine, leucine and isoleucine) and has previously been linked to the development of type 2 diabetes in mice [12]. The authors also noted that more than half of the women in the group later diagnosed with GDM had an elevated BMI (body mass index). Therefore, a correlation between BMI and increased urinary excretion of 2-hydroxyisobutyrate cannot be ruled out. This suggests that abnormalities in amino acid metabolism may be one of the early factors in the pathogenesis of GDM. In the plasma of patients who were later diagnosed with GDM, decreased concentrations of betaine, which is involved in choline metabolism in the homocysteine-to-methionine cycle, as well as trimethylamine *N*-oxide (TMAO), were observed. The results suggest that women, who develop GDM, experience early metabolic disturbances involving amino acid metabolism and choline metabolism [10].

In the latest paper in the series, Díaz et al. [13] focused on assessing the usefulness of the urinary metabolome in predicting pregnancy complications. Although trisomy of 21st chromosome was the main focus of the study, an assessment of metabolic profiles in the context of GDM was also included. In women who went on to develop GDM, an increase in urinary glucose levels was observed; this may be an early sign of developing hyperglycaemia and progressive insulin resistance, appearing even before the standard diagnostic criteria based on the OGTT are met. Furthermore, elevated levels of acetate, niacinamide and pyridine were found in the urine of the study group, along with abnormalities in creatine and creatinine levels. At the same time, decreased concentrations of hippurate and phenylacetylglutamine (PAG) were noted [13].

A combined analysis of the three studies indicates that the impact of GDM on the metabolic profile was moderate, compared with severe foetal developmental pathologies. These studies provided the first evidence that disturbances in maternal carbohydrate metabolism affect the foetus already at an early stage of pregnancy.

A study by Dudzik et al. [14] is one of the first comprehensive metabolomic studies of plasma and urine in women with GDM. In the study group, the only significant difference in urine samples was increased excretion of certain amino acids in women with GDM, which did not show a correlation with the degree of glycaemic control. In contrast, individual plasma metabolite profiles made it possible to clearly distinguish between women with normal glucose tolerance and those with GDM. In the plasma of pregnant women with GDM, the most characteristic feature of the metabolic profile was changes in the concentrations of lysoglycerophospholipids. A significant decrease in the concentrations of lysophosphatidylethanolamine (LPE), lysophosphatidylcholine (LPC) and lysophosphatidylinositol (LPI), particularly those containing long-chain polyunsaturated fatty acids (LC-PUFAs), was demonstrated. In the plasma of patients from the study group, an increased ratio of saturated to unsaturated acyl residues in lysoglycerophospholipids was also observed, accompanied by a decrease in lipoxins and plasmalogens, which have antioxidant properties. This may indicate the presence of chronic inflammation and early disturbances in redox balance [15]. Furthermore, a decrease in sphingomyelin and sphingosine-1-phosphate was observed in women with GDM, with no significant differences in ceramide concentrations. In addition, a more than 30% increase in acetylcarnitine levels was observed, accompanied by a nearly 30% decrease in carnitine and long-chain acylcarnitines. What is more, among the group with GDM, the researchers observed elevated levels of 2-hydroxybutyrate, a metabolite of 2-ketobutyrate, which is considered an early marker of glucose intolerance [16]. At the same time, a reduction in glycine and pyruvate concentrations was observed [14].

A study by López-Hernández et al. [17] examined changes in the urinary metabolome of women with GDM in the third trimester of pregnancy. The study recruited women diagnosed with GDM and healthy pregnant women as a control group, all in their third trimester of pregnancy. Basic clinical parameters, such as blood glucose, creatinine and urea levels, were also monitored. In the serum of women with GDM, a reduction in glucose levels was observed compared with the period prior to the diagnosis of GDM, indicating the effectiveness of the treatment administered. However, an increase in urea and creatinine levels was also observed, suggesting that metabolic abnormalities persisted despite glycaemic control. Metabolomic analysis of urine samples revealed increased concentrations of numerous metabolites in patients with GDM, belonging to various classes of chemical compounds: benzopyrans, carboxylic acids and their derivatives, glycerolipids, indoles, tetrapyrroles, sphingolipids, and steroid derivatives were identified. The presence of certain compounds, such as aspartame, 5-carboxy-α-chromanol, and cucurbitacin C, was interpreted as an effect of dietary changes in patients with GDM as part of non-pharmacological therapy. Increased concentrations of steroid metabolites were also detected in urine samples from women with GDM: 11-oxoandrosterone glucuronide, cortolone-3-glucuronide, tetrahydroaldosterone-3-glucuronide, 5-androstene-3β,16β,17α-triol, and 21-deoxycortisol. Significant changes were also observed in lipid metabolism. Elevated concentrations of glycerolipids, including diacylglycerols, were identified in urine samples from women with GDM; these act as secondary messengers in cells and play a significant role in the mechanisms leading to insulin resistance [18]. In addition, changes in sphingolipid metabolism were observed, including increased concentrations of sphingomyelin SM (d18:0/22:0) and ceramide Cer (d18:0/23:0). A metabolomic analysis of patients with GDM also revealed elevated levels of L-tryptophan [17].

In a study by Herrera-Van Oostdam et al. [19] a metabolomic analysis of urine samples from pregnant women in their second and third trimesters was conducted to assess the impact of GDM on the mother’s metabolic profile and the potential implications of these changes for the newborn’s metabolism. Quantitative analysis did not reveal a clear, distinct metabolomic pattern between women with GDM and the control group in the second and third trimesters, suggesting that metabolic differences between the groups may even out in the later stages of pregnancy. The lack of significant differences may be interpreted as an effect of the implemented treatment, including insulin therapy and lifestyle modifications such as diet and physical activity. These interventions may lead to the normalisation of metabolism in women with GDM, resulting in a metabolomic profile similar to that observed in healthy women. Although there were no clear differences between the groups, significant changes in the concentrations of certain metabolites were observed during pregnancy in both healthy women and those with GDM. In particular, an increase in the concentrations of short-chain fatty acids, such as butyric acid and isobutyric acid, was observed between the second and third trimesters. These compounds are products of intestinal fermentation, and their increased concentrations may reflect changes in the composition and activity of the gut microbiota during pregnancy [20].

One of the first studies to integrate metabolomics with proteomics was a study by Hajduk et al. [21], in which metabolite and plasma protein profiling was carried out in women diagnosed with GDM and in healthy pregnant women. The aim of the study was to identify molecular abnormalities characteristic of GDM and to determine the relationship between changes in the metabolome and proteome. The application of multivariate Partial Least Squares Discriminant Analysis (PLS-DA) demonstrated clear differences between women diagnosed with GDM and those without carbohydrate tolerance disorders, particularly with regard to metabolites such as citrulline, asparagine, ethanolamine, phenylalanine, and serine. It was particularly significant that asparagine and citrulline exhibited both the highest VIP (Variable Importance in Projection) values, which rank variables according to their importance in distinguishing between the study groups, and the lowest *p*-values in the univariate analysis. This strong association between them and the occurrence of GDM was pointed, as well as the potential role of biomarkers of metabolic disorders in the course of the disease. In contrast, proteomic analysis revealed changes in plasma peptide profiles, with particular importance attributed to selected peptide ions of specific m/z values. Identification of one of these ions indicated apolipoprotein A-IV, a protein involved in lipid metabolism and lipoprotein transport [21].

A study by Pinto et al. [22] is one of the key studies on the use of metabolomics to predict the development of GDM, even before its clinical diagnosis. The authors used proton magnetic resonance spectroscopy (^1^H NMR) to analyse the metabolomic profile of blood samples taken from women in their second trimester of pregnancy—both before and after a diagnosis of GDM. Early changes primarily involved an increase in cholesterol levels and a slight rise in the levels of lipoproteins (high-density lipoprotein, low-density lipoprotein, very-low-density lipoprotein), fatty acids and triglycerides, suggesting that lipid metabolism abnormalities may precede the clinical manifestation of the disease. Also, changes in carbohydrate metabolism and energy metabolism, including increased levels of glucose, pyruvate and lactate, were observed. At the same time, an increase in the concentrations of certain amino acids, including glutamine, proline and valine, was noted. With regard to metabolites associated with the gut microbiota, increased concentrations of TMA/TMAO, dimethyl sulphone and methanol were observed. In addition, abnormalities in homocysteine metabolism and the urea cycle were investigated, including changes (mainly increases) in the metabolites of these pathways. The authors also noted that the greatest classification power in the period preceding the diagnosis of GDM, was achieved not for a set of selected individual metabolites, but rather for a full-resolution fragment of the Carr–Purcell–Meiboom–Gill (CPMG) spectrum. Conversely, plasma analysis demonstrated comparable classification accuracy to that of lipid extracts, whilst eliminating time-consuming sample preparation procedures. Following the diagnosis of GDM, metabolic abnormalities became more pronounced, and PLS-DA models enabled the clear differentiation of patients with GDM from healthy pregnant women on the basis of both plasma spectra and lipid extracts. Although some of the changes were partly dependent on gestational age, it was possible to identify a characteristic GDM pattern comprising 26 resonances corresponding to 10 metabolites and lipids. The model based on these signals was characterised by high stability and specificity, comparable to full-resolution spectral analysis.

A landmark step towards the implementation of metabolomics into clinical practice was the development of multimarker diagnostic models for GDM by Hou et al. [23]. The authors did not limit themselves to simply identifying the metabolites that distinguish women with GDM from healthy pregnant women, but focused on developing an integrated diagnostic model that could be put to practical use in risk assessment and the diagnosis of the condition.

The authors conducted a metabolomic analysis of plasma samples from pregnant women using three complementary mass spectrometry platforms, covering different classes of metabolites. The most pronounced changes concerned lipid metabolism, particularly free fatty acids, the blood levels of which were elevated in women with GDM. In addition, elevated levels of BCAAs and changes in bile acid metabolism were observed. The authors suggest that the observed changes may reflect coexisting disorders of glucose, lipid and amino acid metabolism, as well as potential abnormalities within the gut microbiota, which highlights the complex nature of the pathogenesis of GDM. A key part of the work involved developing three diagnostic models, comprising: (1) only metabolites, (2) metabolites in combination with BMI, and (3) metabolites together with BMI and additional clinical markers, including retinol binding protein 4 (RBP4), cystatin C (Cys C) and cholinesterase (ChE). The model containing BMI, RBP4, *N*-acetylaspartic acid, and C16:1 (cis-7) demonstrated the best discriminatory ability, achieving an AUC of 0.751 (95% CI: 0.693–0.809), with a sensitivity of 72.5% and a specificity of 71.7%. The authors emphasised that combining metabolomic data with anthropometric parameters and clinical biomarkers enhances diagnostic value compared with individual indicators.

A study by Odenkirk et al. [24] is one of the most advanced studies to use a multi-omics approach in the analysis of pregnancy complications, including GDM and pre-eclampsia (PE). The aim of the study was to identify the specific molecular signatures of both disease entities. Serum samples from women in their second and third trimesters of pregnancy were analysed, including groups with GDM and PE and healthy pregnant women. The proteomic analysis identified 1093 proteins, 76 of which showed significant differences between the GDM group and the control group. A characteristic feature of GDM was the predominance of decreased protein expression (approximately 76% of significantly altered molecules), particularly among proteins associated with transport, cell adhesion, cholesterol metabolism, and carbohydrate metabolism. Reduced expression of enzymes involved in starch and carbohydrate metabolism, including lysosomal alpha-glucosidase (LYAG) and beta-1,4-galactosyltransferase 1 (B4GT1), as well as proteins associated with β-cell function and glucose homeostasis—glutathione synthetase (GSHB), receptor-type tyrosine-protein phosphatase kappa (PTPRK) and protein/nucleic acid deglycase DJ-1 (PARK7)—points to a molecular basis for carbohydrate metabolism disorders in GDM. Lipidomic analysis included 288 lipids belonging to three main classes: sphingolipids, glycerolipids, and glycerophospholipids. In GDM, 28 significantly altered lipids were identified, with most of them showing reduced concentrations. An exception was phosphatidylethanolamines and their plasmalogen forms (PEP), whose concentrations were elevated. In turn, multi-omics analysis revealed specific correlations between changes in proteins and lipids. Of particular interest was the coexistence of overexpression of selected PEP species containing 22:6 and decreased expression of the phosphatidylethanolamine-binding protein, indicating disturbances in the regulation of signalling pathways and phospholipid metabolism characteristic of GDM. One of the key aspects of the study was a comparison of GDM and PE signatures. Whilst GDM was characterised by predominant energy and lipid abnormalities, PE showed stronger links to sphingolipid metabolism, the activation of inflammatory processes and endothelial dysfunction.

A study by Zhao et al. [25] investigated whether the metabolic abnormalities associated with GDM were already present in early pregnancy and what differences existed between the first and second trimesters. Serum samples were collected from patients who had developed GDM and from healthy pregnant women at two time points, during the first and second trimesters of pregnancy, which enabled an assessment of the dynamic metabolic changes preceding the clinical diagnosis of the condition. In the first trimester, prior to the clinical diagnosis of GDM, 31 metabolites were identified which differed significantly between the study group and the control group, indicating early disturbances in metabolic pathways. In terms of purine metabolism, elevated levels of hypoxanthine and xanthine were observed during the first trimester in women who subsequently developed GDM, along with a more rapid increase in uric acid levels in the subsequent weeks of pregnancy. Additionally, disturbances in the fatty acid β-oxidation pathway were demonstrated in the study group, manifested by elevated concentrations of pantothenic acid and long-chain acylcarnitines already in the first trimester, suggesting early abnormalities in lipid metabolism preceding the diagnosis of the disease. On the other hand, during the second trimester, women with GDM showed decreased concentrations of lysophosphatidylcholines. Analysis of changes over time also revealed significant differences in amino acid metabolism. During the first trimester of pregnancy, elevated levels of proline, leucine, isoleucine, glutamic acid and ornithine were observed among the women in the study group. Furthermore, concentrations of serine and tyrosine were elevated in women with GDM. However, in the second trimester, the concentrations of serine, proline, leucine/isoleucine, glutamic acid, tyrosine and ornithine were significantly lower in the GDM group than in healthy pregnant women. This means that not only the concentration levels but also the rate at which these amino acid concentrations changed over time differed between the groups.

A study conducted by Heath H. et al. [26] aimed to assess the metabolic changes associated with the development of GDM during pregnancy. The analysis included 40 pregnant women who were overweight or obese and participating in a multicentre clinical trial on lifestyle changes. Among the participants, 20 women developed GDM, whilst the remaining 20 formed the control group. Blood plasma samples were taken during the first trimester and the third trimester of pregnancy. Metabolomic analysis revealed significant differences in metabolites, primarily associated with fatty acid metabolism and purine degradation. In the group of women with GDM, a significant decrease in the concentrations of medium-chain acylcarnitines was observed, particularly lauroylcarnitine, octanoylcarnitine, decanoylcarnitine, and decenoylcarnitine. These changes were statistically significant in the analysis of the interaction between the group and time factors (*p* < 0.05), indicating that the pattern of metabolic changes during pregnancy differed between women with GDM and the control group. At the same time, elevated levels of hypoxanthine and inosine monophosphate (IMP) were found in women with GDM. Furthermore, it was demonstrated that during pregnancy, concentrations of bile acids and sorbitol increased in both the study and control group, whereas the levels of numerous acylcarnitines and α-hydroxybutyrate decreased, reflecting the physiological metabolic changes occurring during pregnancy [26].

The authors conclude that the characteristic metabolic profile, characterised by reduced levels of medium-chain acylcarnitines and increased levels of purine degradation metabolites, may reflect impaired fatty acid β-oxidation and changes in energy metabolism in women who develop GDM. The results suggest that these metabolites may serve as potential biomarkers for the pathophysiology of GDM and for its early detection during pregnancy.

Grapov et al. [27] conducted proteomic analysis to investigate the impact of GDM on colostrum composition, with the particular focus on the whey fraction of human milk. The aim of the study was to determine whether the metabolic disorders associated with GDM result in changes to the protein composition of breast milk, which is a key source of nutrients and immune factors for the newborn. The analysis included colostrum samples collected in the first few days after delivery from women with GDM and healthy mothers. Significant changes were observed in proteins associated with lipid metabolism. The concentrations of proteins involved in de novo lipid synthesis were reduced in the colostrum of women with GDM. Importantly, these changes did not correlate with pre-pregnancy BMI, which once again highlights the independent effect of GDM on milk composition. Changes in apolipoprotein levels were also observed. Apolipoprotein D concentrations were elevated in the colostrum of women with GDM, whereas apolipoprotein A1 and apolipoprotein E concentrations were lower. Furthermore, in patients who had GDM during pregnancy, lower concentrations of alpha-2-HS glycoprotein (fetuin-A) and FAM3B (PANDER)—which are associated with carbohydrate metabolism—were observed as compared with those in the control group.

The study’s findings suggest that GDM affects not only the mother’s metabolism during pregnancy, but also the protein composition of colostrum—the newborn’s first food. Furthermore, changes in immune proteins and enzymes involved in lipid metabolism may play a role in the early metabolic and immune programming of the child (Table 2).

## 4. Discussion

In recent years, metabolomics and proteomics have emerged as valuable tools for investigating the pathophysiology of GDM. Unlike conventional diagnostic approaches based primarily on blood glucose measurements and OGTT, omics technologies provide a broader view of metabolic alterations and may detect changes before the clinical onset of GDM [5,6,7].

The studies included in our review consistently demonstrate that GDM is associated with widespread metabolic dysregulation involving lipid, amino acid, energy, and inflammatory pathways. Among the most reproducible findings are disturbances in lipid metabolism, including elevated concentrations of free fatty acids and triglycerides, alterations in phospholipid and sphingolipid composition, and reduced lysophospholipid levels [14,22,23,24]. These changes are biologically plausible, as lipid accumulation, particularly of ceramides and diacylglycerols, may impair insulin signalling and contribute to insulin resistance [17,18]. Similarly, abnormalities in amino acid metabolism, especially increased levels of BCAAs, have been repeatedly linked to insulin resistance and may represent early metabolic markers of disease development [10,23,25]. Alterations in glycine, serine, glutamine, and tryptophan metabolism further suggest interactions between energy homeostasis, oxidative stress, and immune regulation [9,14,22].

Numerous studies have also demonstrated that metabolic changes are detectable during the first trimester of pregnancy, before the clinical diagnosis of GDM [25]. This finding highlights the potential value of metabolomic profiling for risk stratification and early identification of women at increased risk of developing the disease. Moreover, multi-omics approaches integrating metabolomic, proteomic, and lipidomic datasets have revealed complex interactions between metabolic pathways and proteins involved in glucose homeostasis, lipid transport, and inflammatory signalling, emphasising the systemic nature of GDM [21,24]. What is more, omics studies suggest that GDM may have lasting consequences for both mothers and offspring. Women with a history of GDM may exhibit persistent immunometabolic abnormalities after delivery, even when glycaemic control has normalised, potentially contributing to the later development of type 2 diabetes [19,28]. Likewise, analyses of neonatal meconium and urine have identified alterations in metabolites related to amino acid metabolism, fatty acid oxidation, and energy production [20,29]. Furthermore, a study by Herrera-Van Oostdam et al. [19] also analysed the metabolomic profile of the urine of newborns whose mothers had GDM. The analysis employed a targeted quantitative metabolomics approach using gas chromatography coupled with mass spectrometry, which enabled the quantification of 101 metabolites. The results showed that, although most metabolites fell within the reference ranges, newborns of mothers with GDM showed a different metabolic profile, particularly with regard to amino acids, polyamines and carnitines. In the comparative analysis of the two groups, 11 metabolites were identified; however, after correction for multiple comparisons, only two of them remained statistically significant: spermine and acylcarnitine C16:1. The observed increased concentration of spermine in the urine of newborns born to mothers with GDM may indicate disturbances in the arginine/nitric oxide pathway, which plays an important role in vascular endothelial function [30]. Conversely, reduced levels of C16:1 acylcarnitine suggest possible abnormalities in fatty acid metabolism, including β-oxidation, although the mechanism underlying this phenomenon remains unclear [31]. In addition, a tendency towards a reduction in the levels of other long-chain acylcarnitines was observed; however, these changes did not reach statistical significance. These observations support the hypothesis that exposure to a diabetic intrauterine environment may influence early metabolic programming and increase susceptibility to obesity, insulin resistance, and metabolic disease later in life.

Despite the growing number of metabolomic and proteomic studies in GDM, the quality of the available evidence remains heterogeneous. Although the studies included in this review provide valuable insights into the metabolic alterations associated with GDM, considerable variability exists in study design, sample size, analytical validation, and statistical approaches. Most studies were conducted in relatively small cohorts, often including fewer than 100 women with GDM, which limits statistical power and increases the risk of false-positive findings. Furthermore, only a minority of studies performed external validation of identified biomarkers, while systematic assessments of analytical reproducibility and risk of bias were rarely reported. Consequently, the strength of evidence supporting many proposed biomarkers remains limited, and their clinical applicability requires further confirmation in larger, rigorously designed studies.

Another important limitation is the presence of potential confounding factors, including maternal BMI, dietary habits, ethnicity, gestational age, and pharmacological treatment, which are not uniformly controlled for. As a result, some reported metabolic alterations may reflect underlying differences in maternal characteristics rather than GDM pathophysiology. Finally, only a limited number of proposed biomarkers have undergone independent validation in external cohorts, making their clinical utility uncertain. Although the reported findings are promising, they should be interpreted with caution until confirmed in larger, standardised, multicenter studies. These limitations were also evident among the studies included in our review, where considerable differences in sample size, biological matrices analysed, and analytical platforms hindered direct comparison of results and prevented the identification of a universally consistent biomarker panel.

Future studies should adopt standardised approaches to confounder adjustment to improve comparability and strengthen causal interpretation. Another challenge is the predominantly observational design of existing studies, which precludes conclusions regarding causality. It remains unclear whether many of the identified metabolic alterations are causal contributors to GDM pathogenesis or secondary consequences of developing insulin resistance and hyperglycemia. Future longitudinal studies beginning before conception and continuing throughout pregnancy may help clarify these relationships.

Substantial methodological heterogeneity also exists regarding biological samples and analytical techniques. The reviewed studies analysed a wide range of biofluids, including plasma, serum, urine, amniotic fluid, meconium, and colostrum, using different analytical platforms and integrated multi-omics approaches. Since metabolite coverage, sensitivity, quantification accuracy, and sample preparation procedures vary considerably across these methods, direct comparison of findings between studies is challenging. This heterogeneity likely contributes to inconsistencies in reported biomarkers and limits the identification of universally reproducible metabolic signatures of GDM. Interpretation of the findings is further complicated by the fact that studies assessed women at different stages of pregnancy and disease progression. The reviewed literature includes studies aimed at first-trimester prediction of GDM, studies examining metabolic changes before clinical diagnosis during the second trimester, and analyses performed after diagnosis and treatment in later pregnancy. These studies address distinct clinical questions and should not be considered interchangeable. Biomarkers identified during early pregnancy may have predictive value, whereas metabolic alterations observed after diagnosis may primarily reflect established disease processes or treatment effects. Therefore, future reviews and meta-analyses should distinguish more clearly between predictive, diagnostic, and disease-monitoring biomarkers.

Another limitation of our review is that the literature search was restricted to PubMed and Web of Science databases and focused on predefined metabolomics and proteomics related keywords. Consequently, some relevant studies indexed in other databases or using alternative omics terminology may not have been identified.

Although the search strategy included both metabolomic and proteomic terms, the screening process was not restricted to studies applying both approaches simultaneously. Studies investigating either metabolomics or proteomics in GDM were eligible for inclusion provided they met the inclusion criteria. Although our review refers to both metabolomics and proteomics, proteomic studies remain relatively limited and have identified alterations in proteins involved in inflammation, lipid transport, insulin signalling. Nevertheless, the additional clinical value of proteomic profiling beyond metabolomic analysis remains insufficiently established. Direct comparisons between metabolomic and proteomic biomarkers are restricted and it remains unclear whether proteomic markers provide independent predictive information or primarily reflect biological pathways already captured by metabolomic measurements. Future studies integrating both approaches may help determine their complementary roles in understanding GDM pathogenesis and improving risk prediction.

The increasing interest in multi-omics models reflects the recognition that GDM is a complex, systemic disorder involving multiple interacting biological pathways. However, the translation of multi-omics approaches into routine clinical practice faces several practical challenges. Comprehensive metabolomic and proteomic analyses remain expensive, require specialised instrumentation and bioinformatic expertise, and are not yet standardised across laboratories. In addition, issues related to data processing and quality control must be addressed before large-scale implementation becomes feasible. Importantly, it remains unclear how multi-omics-based risk prediction models would be integrated with existing screening strategies based on OGTT and established clinical risk factors. Therefore, future research should not only focus on biomarker discovery but also evaluate cost-effectiveness, clinical utility, and feasibility of implementation.

Future studies should prioritise large multicenter prospective studies with standardised protocols for sample collection, processing, and data analysis. The development of reproducible metabolite panels and validation in independent populations will be essential before omics-based biomarkers can be translated into clinical practice. Further integration of metabolomic, proteomic, genomic, and clinical data, combined with machine learning approaches, may improve risk prediction models and provide deeper insight into the molecular mechanisms underlying GDM. In addition, intervention studies examining the effects of diet, physical activity, microbiome modulation, and pharmacological treatment on metabolomic trajectories could help determine whether these molecular changes are modifiable and clinically meaningful. Such studies may also establish whether metabolomics can serve as a tool for monitoring the effectiveness of preventive and therapeutic interventions.

### Summary

In conclusion, current evidence indicates that GDM is a systemic metabolic disorder characterised by disturbances in lipid, amino acid, energy, and inflammatory pathways. Although metabolomics and proteomics have substantially expanded our understanding of disease mechanisms and identified promising biomarker candidates, methodological heterogeneity, limited sample sizes, and insufficient validation remain major barriers to clinical implementation. Addressing these challenges will be crucial for translating omics discoveries into effective strategies for the early detection, prevention, and management of GDM.

## Figures and Tables

**Table 1:** GDM is diagnosed when any of the results exceed the normal range [4].

Measurement Point	Plasma Glucose Concentration
Fasting	≥5.1 mmol/L
After 1 h	≥10.0 mmol/L
After 2 h	≥8.5 mmol/L

**Table 2:** This table summarises the studies described above, taking into account the analytical techniques used, the biological material examined, and key metabolic changes [9,10,13,14,17,19,21,22,23,24,25,26,27].

Authors	Participants	GDM Diagnostic Criteria	Biological Material	Pregnancy Period	Treatment Ongoing at Sampling	Method	Key Changes	Conclusions
Graça et al. [9]	27 pre-GDM 82 controls	Not reported	Amniotic fluid	15–22 weeks of gestation	No (prediagnostic samples)	Metabolomics	↑ glucose, lactate; ↓ acetate, amino acids	Early metabolic disturbances in amniotic fluid indicate the impact of GDM on the intrauterine environment even before the disease is diagnosed.
Díaz et al. [10]	29 pre-GDM (urine), 19 pre-GDM (plasma) 25 controls (urine), 20 controls (plasma)	Not reported	Urine, plasma	15–22 weeks of gestation	No (prediagnostic samples)	Metabolomics	↑ 3-hydroxyisovalerate; ↓ betaine	Changes in amino acid and choline metabolism may represent some of the earliest signals of GDM development.
Díaz et al. [13]	42 pre-GDM 84 controls	Not reported	Urine	14–26 gestational weeks	Not reported	Metabolomics	↑ glucose; ↓ hippurate	The urinary metabolomic profile may enable non-invasive early detection of GDM before diagnostic criteria are fulfilled.
Dudzik et al. [14]	20 GDM 20 controls	International Association of Diabetes and Pregnancy Study Groups (IADPSG)	Plasma, urine	22–28 weeks of gestation	No	Metabolomics	↓ lysophospholipids; ↑ acylcarnitine	Disturbances in lipid metabolism and redox balance play a key role in the pathogenesis of GDM and may serve as diagnostic targets.
López-Hernández et al. [17]	24 GDM 11 controls	American College of Obstetricians and Gynecologists (ACOG) criteria (OGTT-based; exact diagnostic approach not specified)	Urine	Third trimester	Yes, insulin, metformin or a combination of both	Metabolomics	↑ lipids, steroids	Metabolic disturbances persist despite treatment, indicating their deeper, systemic nature.
Herrera-Van Oostdam et al. [19]	26 GDM 22 controls	ACOG and WHO criteria (OGTT-based; exact diagnostic approach not specified)	Urine	Second–third trimester	Yes, insulin or metformin or a combination of both	Metabolomics	↑ butyric and isobutyric acids	GDM treatment may lead to partial normalisation of the metabolome, making biomarker identification in late pregnancy more challenging.
Hajduk et al. [21]	18 GDM 13 controls	IADPSG	Plasma	24–28th weeks of gestation	Diet treatment	Multi-omics	Changes in amino acids and apolipoprotein A-IV	Integration of metabolomic and proteomic data enables the identification of complex GDM biomarkers with high diagnostic value.
Pinto et al. [22]	32 pre-GDM 12 post-GDM 49 controls	IADPSG	Plasma	Second trimester	No	NMR	↑ lipids, cholesterol	Early alterations in lipid and energy metabolism precede the clinical diagnosis of GDM and may be useful in predictive models.
Hou et al. [23]	131 GMD 138 controls	IADPSG	Plasma	24–28th weeks of gestation	No	Metabolomics	↑ BCAAs	Models combining metabolites with clinical data improve the diagnostic performance of GDM compared to single markers.
Odenkirk et al. [24]	45 GDM 98 controls	Not reported	Serum	third trimester	No	Multi-omics	↓ proteins; lipid alterations	A multi-omics approach reveals the systemic nature of GDM and distinct molecular mechanisms compared with other pregnancy complications.
Zhao et al. [25]	107 GDM 107 controls	IADPSG	Serum	First–second trimester	Not reported	Metabolomics	↑ BCAAs, hypoxanthine	Dynamic metabolomic changes already in the first trimester indicate the possibility of very early identification of GDM risk.
Heath H et al. [26]	20 GDM 20 controls	IADPSG	Plasma	First and third trimester	Not reported	Metabolomics	↓ acylcarnitines	Abnormalities in β-oxidation and purine metabolism reflect the energy disturbances characteristic of GDM.
Grapov et al. [27]	6 GDM 12 controls	Carpenter–Coustan	Colostrum	Postpartum	Yes, insulin, oral antidiabetic medications, diet	Proteomics	↓ apolipoprotein A1; ↑ apolipoprotein D	GDM affects the protein composition of breast milk, which may influence the metabolic and immunological programming of the newborn.

↑ Increase; ↓ decrease.

## Data Availability

The data presented in this study are openly available in PubMed at https://pubmed.ncbi.nlm.nih.gov/?term=%28%22gestational+diabetes+mellitus%22%29+OR+%22gestational+diabetes%22+OR+GDM%29+AND+%28metabolomic*+OR+metabolomics%29+AND+%28proteomic*+OR+proteomics%29&size=50 (accessed on November 2025 until 23 December 2025).
